# Factors influencing adverse reactions to adrenocorticotropic hormone treatment in infantile epileptic spasm syndrome: a single-center retrospective study

**DOI:** 10.3389/fphar.2026.1805530

**Published:** 2026-03-23

**Authors:** Yun-Qing Zhou, Feng Han, Ying-Yan Wang, Xin Zhang, Ji-Wen Wang, Ka-Na Lin, Hao Li

**Affiliations:** 1 Department of Neurology, Shanghai Children’s Medical Center, Shanghai Jiao Tong University School of Medicine, Shanghai, China; 2 Department of Neurology, Hainan Branch, Shanghai Children’s Medical Center, Shanghai Jiao Tong University School of Medicine, Sanya, Hainan, China; 3 Department of Clinical Laboratory Medicine, Shanghai Children’s Medical Center, Shanghai Jiao Tong University School of Medicine, Shanghai, China; 4 Clinical Research Ward, Clinical Research Center, Shanghai Children’s Medical Center, Shanghai Jiao Tong University School of Medicine, Shanghai, China

**Keywords:** ACTH dosing, adrenocorticotropic hormone, adverse events, hypertension, infantile epileptic spasm syndrome, pediatric neurology, respiratory adverse reactions

## Abstract

**Background and Purpose:**

Infantile Epileptic Spasm Syndrome (IESS) is a rare and severe developmental epileptic encephalopathy. Adrenocorticotropic hormone (ACTH) is regarded as one of the three major first-line treatment for IESS. However, the factors associated with the occurrence of adverse reactions during ACTH therapy remain unclear. This study aimed to identify and evaluate the clinical factors associated with the occurrence of adverse events (AEs) in patients with IESS undergoing ACTH therapy.

**Methods:**

This retrospective case-control study included 94 patients with IESS who received ACTH therapy in Shanghai between March 2015 and November 2024. Cases were defined as those who developed clinical AEs. Propensity score matching was employed to minimize potential confounding factors. Association between potential risk factors and AEs were evaluated using the Mann-Whitney U tests and Spearman’s correlation analysis.

**Results:**

Among the study cohort, 24 cases did not experience any AEs. Overall, patients with AEs were associated with a longer duration of ACTH treatment (13.34 vs. 11.79 days, p = 0.074) and a higher total cumulative dose (367.20 vs. 300.29 IU, p = 0.088). In subgroup analyses, respiratory system AEs showed no significant correlation with treatment duration or the cumulative dose. In contrast, the occurrence of hypertension was significantly associated with the daily average dose (25.53 vs. 1.81 IU/day, p = 0.021), the initial dose (22.12 vs. 9.29IU, p < 0.001) and initial average dose (2.73 vs. 1.21 IU/kg, p < 0.001) of ACTH.

**Conclusion:**

Both the duration and cumulative dose of ACTH therapy are positively correlated with the incidence of AEs. The risk of hypertension during ACTH treatment for IESS is significantly linked to the initial dosing of ACTH. Careful consideration should be given to the selection of the initial ACTH dosage before initiating treatment for IESS.

## 1 Introduction

Infantile Epileptic Spasm Syndrome (IESS) is a severe developmental and epileptic encephalopathy characterized by epileptic spasms in clusters, hypsarrhythmia on electroencephalogram (EEG), and neurodevelopmental delay. It typically manifests between 1 and 24 months of age, with a peak incidence at 3–7 months (Zuberi et al., 2022). Without prompt and effective intervention, the condition often progresses to drug-resistant epilepsy and can result in irreversible neurological deficits (Zuberi et al., 2022).

Adrenocorticotropic hormone (ACTH) is recommended by the International League Against Epilepsy (ILAE) as one of the three major first-line therapy for IESS, the others being oral corticosteroids and vigabatrin (Zuberi et al., 2022). Regarding its dosage, findings from the American Academy of Neurology and the Child Neurology Society indicate comparable efficacy between low-dose (20–30 IU/day) and high-dose (150 IU/m^2^/day) ACTH regimens (Go et al., 2012). This is supported by a retrospective analysis which found no significant difference in short-term response rates between high-dose (120 IU/day) and low-to-moderate-dose ACTH (40 IU/day) ACTH (Riikonen et al., 2020). However, a meta-analysis presents a contrasting perspective, showing that high-dose ACTH (>30 IU/day) was associated with significantly higher rates of complete spasm cessation compared to low-dose regimens (<30 IU/day), albeit with an elevated risk of hypertension (Devi et al., 2024).

The safety profile of ACTH, particularly concerning hypertension, is a significant clinical concern. A single-center retrospective study in the United States reported hypertension in 44% of children receiving ACTH therapy (McGarry et al., 2020), while a study in India observed this adverse effect in 93.3% of cases (Angappan et al., 2019). The striking discrepancy in hypertension rates is primarily driven by two key factors: ethnic differences in physiological responsiveness to ACTH, and variations in ACTH formulation and dosing regimens. Such variability underscores the necessity of population-specific safety data for guiding clinical practice. Beyond hypertension, ACTH treatment is also associated with increased susceptibility to infection and emotional lability (Devi et al., 2024; Angappan et al., 2019; Grinspan et al., 2021).

Despite these insights, there remains a paucity of data specifically illustrating the relationship between ACTH dosage and treatment safety within the Chinese pediatric population. To address this gap, we conducted the present retrospective study to explore the potential factors influencing adverse reactions to ACTH Treatment in Chinese patients with IESS.

## 2 Methods

### 2.1 Study design and setting

This was a retrospective case-control study conducted in accordance with the Strengthening the Reporting of Observational Studies in Epidemiology (STROBE) guidelines (von et al., 2007). The study was carried out at Shanghai Children’s Medical Center, affiliated with Shanghai Jiao Tong University School of Medicine, from March 2015 to November 2024. The study protocol, including all informed consent documents and research procedures, was reviewed and approval by the Ethics Committee of Shanghai Children’s Medical Center (approval number: SCMCIRB-K2023015-1).

### 2.2 Participants and data sources

Patients diagnosed with IESS and treated with natural porcine ACTH (corticotropin, 39-amino-acid polypeptide, manufactured by Shanghai First Biochemical Pharmaceutical Co., Ltd., China) were enrolled in this study. The diagnostic of IESS was based on the 2022 ILAE classification of epilepsy syndromes (Zuberi et al., 2022). Both cases and control participants were required to meet the following inclusion criteria: (1) age between 1 month and 24 months and (2) receipt of ACTH therapy for more the 7 days during hospitalization.

Exclusion criteria were: (1) previous hormone therapy or concurrent use of vigabatrin prior to ACTH treatment; (2) comorbid tuberous sclerosis complex; (3) diagnosis of hypothyroidism; (4) receipt of immunosuppressive therapy following liver transplantation; (5) evidence of infection prior the ACTH initiation, defined as white blood cells count above the normal range; (6) abnormal liver function before ACTH treatment, indicated by elevated liver enzymes; or (7) abnormal serum electrolytes levels, such as hypernatremia or hyperkalemia prior to therapy initiation.

Clinical data were extracted from the hospital’s electronic medical record system. A structured electronic database was created to collect information across the following domains: demographic and general information, including sex, age, height, weight, and birth history; auxiliary examinations findings, including video electroencephalogram (VEEG) and cranial MRI results; IESS-specific data, covering age at seizure onset and interval from onset to seizure frequency assessment; ACTH treatment details, including the dosage and treatment duration; concomitant medications administered during ACTH therapy; clinical efficacy assessment; and documentation of adverse events (AEs).

### 2.3 Study cohorts and variables

In this case-control study, cases were defined as patients who developed any adverse event during ACTH treatment, while controls comprised those who did not experience any adverse event.

The following exposure variables were examined in relation to ACTH treatment outcomes: sex, age, height, weight, birth history (preterm infant at < 37 weeks, birth weight <2500 g, breastfeeding history, neonatal brain injury), age at spasms onset, interval from seizure onset to assessment, seizure frequency ≤5 per day, ACTH treatment parameters (total cumulative dose, cumulative dose per body weight, initial dose, initial average daily dose).

All patients received ACTH at varying therapeutic doses. Based on the median cumulative dose of the entire cohort which were rounded to the nearest whole number, patients were classified into high- and low-dose groups. The low-dose group was defined as receiving a total cumulative dose ≤325 IU and a cumulative dose per kilogram ≤35 IU/kg, whereas the high-dose group received >325 IU and >35 IU/kg.

Initial ACTH doses were administered as 3.13, 6.25, 8.33, 12.5, or 25 IU. Due to only six patients receiving ≤8.33 IU initially, the low initial-dose group was redefined as ≤ 12.5 IU, and the high initial-dose group as 25 IU.

The association between ACTH dose exposure and AE occurrence was evaluated. AEs of interest included:

Respiratory tract adverse reactions: symptoms suggestive of respiratory infections, such as cough, sputum production, or wheezing.

Hypertension: Blood pressure was measured before and during ACTH therapy using an aneroid sphygmomanometer. Hypertension was diagnosis based on age-, sex-, and height-specific percentiles, with systolic or diastolic pressure ≥95th percentile recorded as an event.

### 2.4 Subgroup analysis

Subgroup analyses were performed to identify exposure factors specifically associated with the two most clinically significant adverse event categories observed in this cohort: respiratory tract adverse reactions and hypertension. For each adverse event subgroup, cases were defined as patients who developed the specific event, while controls comprised all other patients, including those with other or no AEs. Within each subgroup, the associations between the event occurrence and the predefined exposure variables were assessed using the same statistical methods applied in the primary analysis. This approach aimed to elucidate whether distinct risk factor profiles existed for different types of treatment-related adverse outcomes.

### 2.5 Statistical analysis

Statistical analyses were conducted using SPSS (Version 26; IBM Corp.). To eliminate the confounding effects arising from differences in baseline characteristics between the case and control groups, we performed propensity score matching (PSM) to balance the distribution of covariates between the two groups. Continuous variables were presented as mean ± standard deviation if normally distributed, and group comparisons (case vs. control) were performed using independent sample t-tests and analysis of variance (ANOVA). For non-normally distributed continuous data, results were expressed as median (interquartile range), and the Mann-Whitney *U* test was used for between-group comparisons. Categorical variables were summarized as frequencies and percentages, and differences were assessed using the Chi-square tests or Fisher’s exact test as appropriate. The Spearman rank correlation coefficient was used to evaluate the association between continuous ACTH doses parameters and short-term treatment outcomes.

## 3 Results

### 3.1 Factors influencing ACTH therapeutic safety

**FIGURE 1:**
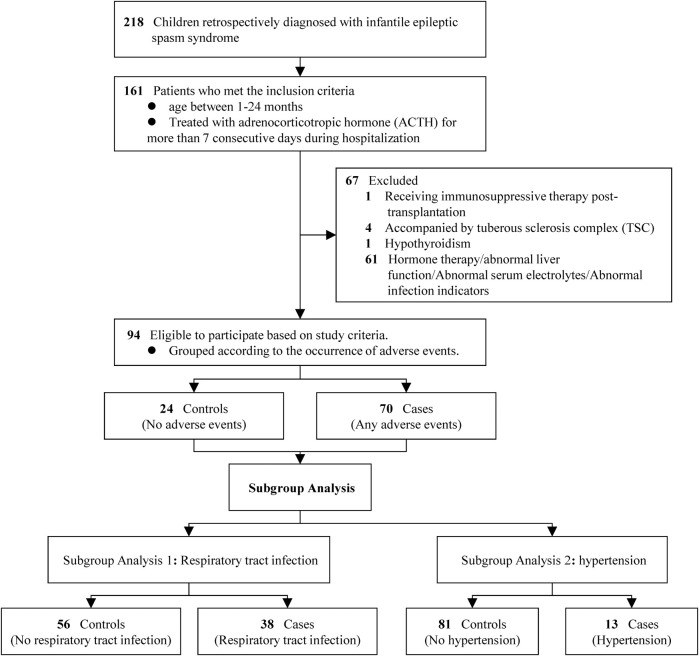
Study flowchart.

During the study period (March 2015 to November 2024), a total of 94 patients met the eligibility criteria and were included in the analysis. Based on the occurrence of AEs, they were categorized into a case group (patients who experienced any AE, n = 70) and a control group (patients without any AE, n = 24) (Figure 1). As summarized in Table 1, the two groups were comparable at baseline, with no statistically significant differences in sex, age, height, weight, birth history, seizure frequency, or interval from seizure onset to treatment initiation (all P > 0.05).

**TABLE 1:** Characteristics of cases and controls.

Index	Cases (*n* = 70)	Controls (*n* = 24)	*P* value
Sex, male/female	40/30	14/10	1.000
Age (range), months	10.95 ± 5.63 (2.73–23.73)	10.02 ± 4.21 (4.13–18.73)	0.687
Height (range), cm	73.38 ± 7.91 (59.00–98.00)	71.36 ± 7.07 (53.00–80.50)	0.535
Weight (range), kg	9.19 ± 2.04 (5.40–14.50)	8.97 ± 1.08 (5.90–11.00)	0.696
Body surface area (range), m^2^	0.42 ± 0.08 (0.25–0.59)	0.41 ± 0.04 (0.29–0.61)	0.696
Onset age of spasms (%), months	​	​	0.911
≤2	17 (24.20)	5 (20.80)	​
3–5	21 (30.00)	9 (37.50)	​
6–11	23 (32.90)	8 (33.30)	​
12–24	9 (12.90)	2 (8.30)	​
*T*_ot_, (range), months	4.70 ± 4.35 (0.27–17.30)	4.85 ± 3.78 (0.23–12.07)	0.742
Birth history (%)	​	​	​
Preterm infant at < 37 weeks	7 (10.00)	3 (12.50)	0.676
Birth weight <2,500 g	5 (7.10)	3 (12.50)	0.675
Breastfeeding	48 (68.60)	12 (50.00)	0.140
Neonatal brain injury history	40 (57.10)	12 (50.00)	0.636
Seizure frequency ≤5/day (%)	44 (62.90)	15 (62.50)	1.000

Results were presented as Mean ± SD., data in the table were obtained before the patient received adrenocorticotropic hormone treatment.

Abbreviations: *T*_ot_, time from spasms onset to adrenocorticotropic hormone treatment.

In the study of ACTH-related over-safety risk, no statistical differences were observed in inflammatory and nutritional indices (e.g., neutrophil/lymphocyte percentage, neutrophil/lymphocyte ratio, prealbumin) between the cases and controls before ACTH treatment (all P > 0.05, Table 2). During ACTH treatment, the treatment duration and total cumulative dose of the cases were higher than those of the controls, approaching statistical significance with P = 0.074 and 0.088, respectively, while no significant differences were found in other treatment indices such as daily average dose and initial dose between the two groups (all P > 0.05, Table 2).

**TABLE 2:** Comparative risks of overall safety.

Index	Cases (*n* = 70)	Controls (*n* = 24)	*P* value
Before ACTH treatment			
Percentage of neutrophils (%)	29.53 ± 11.98 (5.90–63.30)	25.88 ± 8.59 (12.30–45.90)	0.169
Percentage of lymphocytes (%)	59.51 ± 12.56 (27.80–77.90)	63.50 ± 9.02 (44.00–78.50)	0.220
Neutrophils/lymphocytes	0.58 ± 0.45 (0.08–2.27)	0.43 ± 0.22 (0.16–1.05)	0.103
Pre albumin (g/L)	0.22 ± 0.06 (0.10–0.39)	0.23 ± 0.06 (0.13–0.37)	0.451
Neutrophils/lymphocytes >0.5	24 (34.30)	6 (25.00)	0.457
Pre albumin > the normal value	45 (64.30)	17 (70.80)	0.891
ACTH treatment			
Course of treatment, days	13.34 ± 4.45 (7.00–27.00)	11.79 ± 4.90 (7.00–28.00)	0.074
Total cumulative dose, (range), IU	367.20 ± 166.35 (87.49–725.00)	300.29 ± 137.91 (125.00–552.50)	0.088
Daily average dose, (range), IU/d	27.23 ± 8.56 (10.78–44.23)	25.57 ± 7.34 (11.90–40.00)	0.377
Daily average weight dose, (range), IU/d/kg	3.06 ± 1.03 (1.30–5.42)	2.89 ± 0.88 (1.27–4.62)	0.752
Initial dose, (range), IU	18.27 ± 6.94 (6.25–25.00)	16.84 ± 7.29 (6.25–25.00)	0.358
Initial average dose, (range), IU/kg	2.07 ± 0.87 (0.60–3.97)	1.92 ± 0.92 (0.66–4.24)	0.555

Results were presented as Mean ± SD., Abbreviations; ACTH, adrenocorticotropic hormone.

### 3.2 Subgroup analysis of clinical factors influencing respiratory adverse events

To elucidate the clinical factors contributing to respiratory adverse events in this cohort, we first performed a prespecified subgroup analysis evaluating the risk correlates of these events. Table 3 summarizes the baseline demographic, clinical, and perinatal characteristics of cases (n = 38) and controls (n = 56) in this subgroup analysis. Across all measured parameters, including sex distribution, age, anthropometric metrics, spasm onset age, time from spasm onset to ACTH initiation (*T*_ot_), perinatal history, neonatal brain injury history, and seizure frequency, no statistically significant differences were observed between the two groups (all P > 0.05). These results confirm that the cases and controls were well balanced at baseline, thereby mitigating baseline confounding and strengthening the validity of subsequent comparative analyses.

**TABLE 3:** Baseline characteristics of cases and controls in relation to respiratory adverse events.

Index	Cases (*n* = 38)	Controls (*n* = 56)	P value
Sex, male/female	23/15	31/25	0.619
Age (range), months	11.37 ± 5.95 (2.73–23.30)	10.35 ± 4.81 (3.33–23.73)	0.614
Height (range), cm	73.56 ± 7.58 (61.00–90.00)	72.59 ± 7.82 (53.00–98.00)	0.850
Weight (range), kg	9.40 ± 2.01 (6.00–14.00)	9.00 ± 1.71 (5.40–14.50)	0.464
Body surface area (range), m^2^	0.43 ± 0.07 (0.31–0.59)	0.41 ± 0.06 (0.29–0.61)	0.464
Onset age of spasms (%), months	​	​	0.600
≤2	11 (28.90)	11 (19.60)	​
3–5	12 (31.60)	18 (32.10)	​
6–11	10 (26.30)	21 (37.50)	​
12–24	5 (13.20)	6 (10.70)	​
*T*_ot_, (range), months	5.32 ± 4.47 (0.27–15.43)	4.46 ± 4.06 (0.23–17.30)	0.378
Birth history (%)			
Preterm infant at < 37 weeks	5 (13.20)	5 (8.90)	0.249
Birth weight <2,500 g	3 (7.90)	5 (8.90)	0.858
Breastfeeding	27 (71.10)	33 (58.90)	0.277
Neonatal brain injury history	20 (52.60)	32 (57.10)	0.679
Seizure frequency ≤5/day (%)	26 (68.40)	33 (58.90)	0.391

Results were presented as Mean ± SD., data in the table were obtained before the patient received adrenocorticotropic hormone treatment.

Abbreviations: *T*_ot_, time from spasms onset to adrenocorticotropic hormone treatment.

Table 4 compares inflammatory, nutritional, and ACTH treatment-related variables between patients with and without respiratory adverse events. Prior to ACTH initiation, no significant intergroup differences were detected in neutrophil percentage, lymphocyte percentage, neutrophil-to-lymphocyte ratio, or prealbumin concentrations (all P > 0.05). Similarly, the proportions of patients with a neutrophil-to-lymphocyte ratio >0.5 or prealbumin levels above the normal reference range were comparable between groups (P = 0.822 and P = 0.221, respectively). With respect to ACTH treatment parameters, including treatment duration, total cumulative dose, daily average dose, weight-based daily dose, initial dose, and weight-based initial dose, no statistically significant differences were identified between cases and controls (all P > 0.05). Collectively, these findings indicate that the assessed inflammatory, nutritional, and treatment-related factors did not differ significantly between patients who developed respiratory adverse events and those who did not.

**TABLE 4:** Comparative risks of respiratory adverse events.

Index	Cases (*n* = 38)	Controls (*n* = 56)	P value
Before ACTH treatment			
Percentage of neutrophils (%)	30.26 ± 11.61 (12.40–63.30)	27.50 ± 11.00 (5.90–61.70)	0.261
Percentage of lymphocytes (%)	59.47 ± 12.57 (27.80–77.90)	61.24 ± 11.38 (30.80–78.50)	0.467
Neutrophils/lymphocytes	0.60 ± 0.47 (0.15–2.27)	0.51 ± 0.36 (0.08–2.00)	0.222
Pre albumin (g/L)	0.21 ± 0.05 (0.11–0.32)	0.23 ± 0.06 (0.10–0.39)	0.227
Neutrophils/lymphocytes >0.5	13 (34.20)	17 (30.40)	0.822
Pre albumin > the normal value	23 (60.50)	39 (69.60)	0.221
ACTH treatment			
Course of treatment	13.75 ± 5.33 (7.00–27.00)	12.39 ± 4.10 (7.00–28.00)	0.321
Total cumulative dose, (range), IU	351.06 ± 151.24 (87.49–657.50)	347.29 ± 171.11 (103.66–725.00)	0.633
Daily average dose, (range), IU/d	26.20 ± 8.73 (10.78–44.23)	27.18 ± 7.99 (11.90–41.67)	0.751
Daily average weight dose, (range), IU/d/kg	2.83 ± 0.94 (1.30–5.24)	3.11 ± 1.03 (1.27–5.42)	0.196
Initial dose, (range), IU	17.25 ± 7.30 (6.25–25.00)	18.08 ± 6.88 (6.25–25.00)	0.756
Initial average dose, (range), IU/kg	1.89 ± 0.85 (0.60–3.73)	2.08 ± 0.88 (0.66–4.24)	0.434

Results were presented as Mean ± SD., Abbreviations; ACTH, adrenocorticotropic hormone.

### 3.3 Subgroup analysis of clinical factors influencing hypertensive events

To explore the clinical factors associated with hypertensive events, we conducted a prespecified subgroup analysis of patients who developed these events and matched controls. Table 5 presents the baseline demographic, clinical, and perinatal characteristics before and after propensity score matching (PSM). Prior to PSM, statistically significant imbalances were observed between cases (n = 13) and controls (n = 81) in key baseline parameters, including age (P = 0.032), weight (P = 0.088), body surface area (P = 0.088), and the interval from spasm onset to ACTH treatment (*T*_ot_, P = 0.066). To mitigate these baseline confounding effects, we performed 1:1 PSM, which successfully balanced all measured baseline characteristics between the matched case (n = 13) and control (n = 13) groups (all P > 0.05), thereby enhancing the validity of subsequent comparative analyses.

**TABLE 5:** Baseline characteristics of cases and controls in relation to hypertensive events.

Characteristics	Before PSM		P value	After PSM		P value
	Cases (*n* = 13)	Controls (*n* = 81)		Cases (*n* = 13)	Controls (*n* = 13)	
Sex, male/female	7/6	47/34	1.000	7/6	7/6	1.000
Age, (range), months	7.76 ± 3.66 (3.33–15.20)	11.24 ± 5.36 (2.73–23.73)	0.032	7.76 ± 5.88 (3.33–15.20)	8.02 ± 4.40 (3.17–16.47)	0.870
Height, (range), cm	69.65 ± 5.05 (61.00–78.00)	73.51 ± 7.95 (53.00–98.00)	0.091	69.65 ± 5.05 (61.00–78.00)	70.46 ± 7.70 (59.00–83.00)	0.755
Weight (range), kg	8.26 ± 1.62 (5.50–11.00)	9.30 ± 1.83 (5.40–14.50)	0.088	8.26 ± 1.61 (5.50–11.00)	8.20 ± 1.71 (5.40–11.00)	0.926
Body surface area (range), m^2^	0.39 ± 0.06 (0.29–0.49)	0.43 ± 0.06 (0.29–0.61)	0.088	0.39 ± 0.06 (0.29–0.49)	0.39 ± 0.06 (0.29–0.49)	0.926
Onset age of spasms (%), months	​	​	0.786	​	​	0.779
≤2	4 (30.80)	18 (22.20)	​	4 (30.80)	4 (30.80)	​
3–5	5 (38.40)	25 (30.90)	​	5 (38.40)	6 (46.10)	​
6–11	3 (23.10)	28 (34.60)	​	3 (23.10)	3 (23.10)	​
12–24	1 (7.70)	10 (12.30)	​	1 (7.70)	0 (0)	​
*T*_ot_, (range), months	2.73 ± 2.78 (0.27–8.13)	5.14 ± 4.34 (0.23–17.30)	0.066	2.73 ± 2.78 (0.27–8.13)	3.77 ± 3.20 (0.27–9.273)	0.387
Birth history (%)						
Preterm infant at < 37 weeks	0 (0)	10 (12.30)	0.504	0 (0)	2 (15.40)	0.183
Birth weight <2,500 g	1 (7.70)	7 (8.60)	1.000	1 (7.70)	0 (0)	0.593
Breastfeeding	8 (61.50)	52 (64.20)	1.000	8 (61.50)	8 (61.50)	1.000
Neonatal brain injury history	7 (53.80)	45 (55.60)	1.000	7 (53.80)	8 (61.50)	1.000
Seizure frequency ≤5/day (%)	6 (46.20)	53 (65.40)	0.222	6 (46.20)	7 (53.80)	1.000

Results were presented as Mean ± SD., data in the table were obtained before the patient received adrenocorticotropic hormone treatment.

Abbreviations: *T*_ot_, time from spasms onset to adrenocorticotropic hormone treatment, PSM, propensity score matching.

Table 6 compares inflammatory, nutritional, and ACTH treatment-related indices between patients with and without hypertensive events, both before and after PSM. Prior to matching, pre-treatment inflammatory indices demonstrated statistically significant differences: the percentage of neutrophils was lower in cases (22.02% ± 9.57%) than in controls (29.66% ± 11.21%, P = 0.038), while the percentage of lymphocytes was higher in cases (66.36% ± 8.63%) than in controls (59.59% ± 12.05%, P = 0.065), and the neutrophil-to-lymphocyte ratio was also lower in cases (0.36 ± 0.20 vs. 0.57 ± 0.43, P = 0.052). Following PSM, these inflammatory and nutritional parameters were well balanced between groups (all P > 0.05). In contrast, ACTH treatment dosing emerged as a key correlate of hypertensive events: after PSM, significant differences were observed in the initial dose (P < 0.001), initial weight-based dose (P < 0.001), and daily average dose (P = 0.021), with cases receiving higher doses across these metrics. Collectively, these findings highlight that ACTH dosing parameters, particularly the initial dose, are the primary factors associated with an increased risk of hypertensive events in this cohort.

**TABLE 6:** Comparative risks of hypertensive events.

Index	Before PSM		*P* value	After PSM		*P* value
	Cases (*n* = 13)	Controls (*n* = 81)		Cases (*n* = 13)	Controls (*n* = 13)	
Before ACTH treatment						
Percentage of neutrophils (%)	22.02 ± 9.57 (5.90–40.20)	29.66 ± 11.21 (12.60–63.30)	**0.038**	22.02 ± 9.57 (5.90–40.20)	24.45 ± 9.14 (12.40–45.50)	0.515
Percentage of lymphocytes (%)	66.36 ± 8.63 (48.50–77.60)	59.59 ± 12.05 (27.80–78.50)	0.065	66.36 ± 8.63 (48.50–77.60)	65.69 ± 10.48 (42.90–77.90)	0.860
Neutrophils/lymphocytes	0.36 ± 0.20 (0.08–0.83)	0.57 ± 0.43 (0.15–2.27)	0.052	0.36 ± 0.20 (0.08–0.83)	0.40 ± 0.25 (0.15–1.06)	0.604
Pre albumin (g/L)	0.21 ± 0.07 (0.10–0.36)	0.22 ± 0.05 (0.11–0.39)	0.263	0.21 ± 0.07 (0.10–0.36)	0.22 ± 0.0 (0.11–0.32)	0.753
Neutrophils/lymphocytes >0.5	2 (15.40)	28 (34.60)	0.213	2 (15.40)	2 (15.40)	1.000
Pre albumin > the normal value	11 (84.60)	51 (63.00)	0.414	11 (84.60)	10 (76.90)	1.000
ACTH treatment						
Course of treatment	13.85 ± 3.60 (10.00–22.00)	12.77 ± 4.79 (7.00–28.00)	0.272	13.85 ± 3.60 (10.00–22.00)	15.77 ± 7.73 (8.00–28.00)	0.427
Total cumulative dose, (range), IU	358.08 ± 169.77 (175.00–675.00)	347.23 ± 162.67 (87.49–725.00)	0.882	358.08 ± 169.77 (175.00–675.00)	271.93 ± 125.83 (87.49–535.40)	0.155
Daily average dose, (range), IU/d	25.53 ± 9.03 (13.46–41.67)	27.00 ± 8.16 (10.78–44.23)	0.398	25.53 ± 9.03 (13.46–41.67)	18.10 ± 6.07 (10.78–29.38)	**0.021**
Daily average weight dose, (range), IU/d/kg	3.14 ± 1.10 (1.61–5.24)	2.98 ± 0.99 (1.27–5.42)	0.904	3.14 ± 1.10 (1.61–5.24)	2.34 ± 1.11 (1.30–4.90)	0.079
Initial dose, (range), IU	22.12 ± 5.48 (12.50–25.00)	17.04 ± 7.01 (6.25–25.00)	**0.020**	22.12 ± 5.48 (12.50–25.00)	9.29 ± 2.77 (6.25–12.50)	< **0.001**
Initial average dose, (range), IU/kg	2.73 ± 0.75 (1.33–3.85)	1.88 ± 0.83 (0.60–4.24)	**0.003**	2.73 ± 0.75 (1.33–3.85)	1.21 ± 0.55 (0.60–2.31)	< **0.001**

Results were presented as Mean ± SD., Abbreviations; ACTH, adrenocorticotropic hormone; PSM, propensity score matching. Bold values indicate P < 0.05, representing statistical significance.

## 4 Discussion

ACTH is established as a first-line therapy for IESS, however, its optimal regimen remains a subject of international debate, driven by the imperative to balance clinical efficacy, adverse event risk, and economic considerations (Riikonen et al., 2020; Abu et al., 2020; Ramantani et al., 2022). Documented adverse effects include hypertension, increased appetite, irritability, respiratory infections, gastrointestinal infections, hypokalemia, and cardiac symptoms such as arrhythmia, among others (Devi et al., 2024; Ramantani et al., 2022; Hamano et al., 2006). In this retrospective case-control study, we systematically examined the relationship between ACTH dosing parameters and safety outcomes, with a focus on respiratory AEs and hypertension.

Prior studies have consistently linked ACTH-related AEs in IESS to longer treatment duration and higher cumulative doses (Hrachovy et al., 1994). A systematic review and meta-analysis demonstrated that high-dose ACTH was associated with a significantly higher risk of AEs, with cumulative dose being a key determinant of this elevated risk (Guang et al., 2022). Our primary analysis of overall safety risks revealed that cases experiencing AEs tended to have longer treatment courses and higher cumulative doses, with these differences approaching statistical significance (P = 0.074 and 0.088, respectively). While our findings did not reach formal statistical significance, this is likely attributable to multiple factors: first, the relatively modest sample size (n = 94) and unequal group distribution (cases n = 70 vs. controls n = 24), which may have limited statistical power to detect subtle but clinically meaningful differences; second, potential unmeasured confounding factors (e.g., concurrent medication use, variability in supportive care) that could have masked the true association between dosing and AE risk; and third, the heterogeneous nature of AEs included in the primary analysis (encompassing respiratory events, hypertension, and other toxicities), which may have diluted the signal for dose-dependent effects specific to individual AE subtypes. Despite the lack of statistical significance, this trend aligns with the well-documented dose-dependent toxicity of ACTH and reinforces the clinical principle that minimizing exposure duration and cumulative dose may reduce adverse event risk.

Notably, no evidence has linked respiratory adverse events to ACTH dose dependency in the existing literature (Ramantani et al., 2022; Guang et al., 2022). A retrospective analysis of 211 infants with IESS found that upper respiratory tract infections occurred significantly more frequently in those receiving ACTH via the intravenous route compared to the intramuscular route, suggesting administration route may be an underrecognized risk factor (Wu et al., 2024). In our subgroup analysis of respiratory adverse events, we found no significant differences in baseline characteristics, pre-treatment inflammatory/nutritional indices, or ACTH dosing parameters between patients with and without these events. This lack of identifiable risk factors is primarily attributable to our study’s inclusion criteria, which explicitly excluded patients with overt pre-existing infection risks or significant hepatic/renal dysfunction prior to ACTH initiation. By excluding these high-risk individuals, we may have eliminated the primary clinical drivers of respiratory adverse events, thereby reducing variability in the remaining cohort and limiting statistical power to detect associations with the measured variables. Additionally, respiratory AEs may be influenced by factors not captured in our analysis, such as concurrent viral/bacterial infections, underlying pulmonary comorbidities, or individual differences in immune response to ACTH-induced immunosuppression. Future studies with broader inclusion criteria that incorporate patients with pre-existing infections or organ dysfunction are needed to better delineate the risk factors for respiratory AEs in IESS.

It is well established that hypertension represents a definitive dose-dependent adverse effect of ACTH therapy for IESS (Ramantani et al., 2022; Guang et al., 2022). A randomized controlled trial comparing high-dose, long-duration versus low-dose, short-duration ACTH therapy found that hypertension occurred more frequently in the high-dose, long-duration group, with a clear dose-dependent trend in the incidence of systemic AEs (Hrachovy et al., 1994). In our study, hypertension was observed in 13 patients (13.8%), which was consistent with the incidence reported by Yin et al. (Yin et al., 2017). In contrast, our analysis of hypertensive events yielded more definitive findings, with several key observations aligning with and extending our prior case report (Li et al., 2020). Prior to PSM, we observed significant baseline imbalances in age, weight, and treatment latency, which could have confounded our results. After 1:1 PSM, these baseline differences were eliminated, allowing us to isolate the impact of ACTH dosing. Our results clearly demonstrate that higher initial doses (both absolute and weight-based) and higher daily average doses are independent risk factors for hypertension. This is consistent with the known mechanism of ACTH-induced adrenal hyperstimulation, which increases cortisol secretion and triggers sodium retention and vasoconstriction in the highly sensitive infant vasculature (McGarry et al., 2020). Notably, we also observed pre-matching differences in inflammatory indices (lower neutrophil percentage, higher lymphocyte percentage, and lower neutrophil-to-lymphocyte ratio in cases), which were eliminated after PSM—suggesting these variations may be confounded by baseline disparities rather than representing definitive evidence of a direct causal relationship with hypertension. While our current analysis cannot rule out potential associations between these inflammatory markers and the development of hypertension, their specific role as contributing factors or mediating variables requires further dedicated investigation to validate and clarify the underlying mechanisms.

This study has both strengths and limitations that warrant careful consideration. The principal strength of this study resides in its analysis of the correlation between different ACTH administration strategies and the safety of IESS treatment, thus offering a valuable reference for the development of future ACTH treatment protocols for IESS. However, this study also presents several notable limitations. First, the retrospective study design carries a significant risk of confounding bias, such as unaccounted for drug interactions. Secondly, the modest sample size and the absence of long-term follow-up are concerning. Thirdly, the use of median-based dose grouping may obscure non-linear dose-response relationships. Fourthly, there is inadequate laboratory monitoring, for example, regarding the recovery of hypothalamic-pituitary-adrenal axis function post-therapy and biomarkers related to therapeutic efficacy (Lin et al., 2025). Fifthly, the present study was restricted to patients receiving ACTH monotherapy and did not include comparative data with the other two first-line treatments for IESS, namely, oral corticosteroids and vigabatrin. As such, we were unable to directly compare the safety profiles and adverse event risks across the three standard first-line therapies. Future prospective studies are warranted to evaluate and contrast the safety, tolerability, and optimal dosing strategies of ACTH, oral corticosteroids, and vigabatrin in the management of IESS.

In conclusion, this study elucidated the association between ACTH dosing parameters and safety outcomes in IESS, focusing on respiratory AEs and hypertension. We confirmed hypertension as a definitive dose-dependent adverse effect of ACTH therapy, with higher initial and daily average doses identified as independent risk factors. No significant link was found between ACTH dosing and respiratory AEs, a finding likely influenced by our strict inclusion criteria excluding high-risk patients and unmeasured confounding factors. While pre-matching differences in inflammatory indices were resolved by PSM, their potential association with hypertension cannot be excluded and merits further dedicated investigation. Collectively, our results highlight the value of optimized low-dose ACTH regimens for reducing hypertension risk in IESS and call for broader, mechanism-focused studies to further clarify the risk factors for respiratory AEs and the role of inflammatory markers in ACTH-related adverse events.

## Data Availability

The raw data supporting the conclusions of this article will be made available by the authors, without undue reservation.
